# The prevalence and influencing factors of coexisting prediabetes and prehypertension among Bangladeshi adults

**DOI:** 10.1186/s12889-023-16090-z

**Published:** 2023-06-20

**Authors:** Maksuda Yesmin, Masum Ali, Sanjib Saha

**Affiliations:** 1 Health Economics Unit, Department of Clinical Science (Malmö), Lund University, Medicon Village, Scheelevagen 2, 223 63 Lund, Sweden; 2 International Food Policy Research Institute (IFPRI), Dhaka, Bangladesh

**Keywords:** Bangladesh, Prediabetes, Prehypertension, NCDs, Body mass index

## Abstract

**Background:**

Early detection of diabetes and hypertension is helpful to prevent and/or delay the onset of these diseases through proper interventions. Therefore, it is a prerequisite to know the prevalence of prediabetes and prehypertension and the factors associated with these conditions but people from developing countries including Bangladesh often remain undiagnosed and unaware of these conditions. In this study we investigate the prevalence of prediabetes and prehypertension and their associated factors in Bangladesh using nationally representative data.

**Method:**

We used nationally representative Bangladesh Demographic and Health Survey (BDHS) 2017–18 survey data, which included a total sample of 14,704 adults aged 18 years and more from whom blood pressure and fasting plasma glucose were collected. Chi-square test was used to examine the differences between sociodemographic and outcome variables. The univariate and multivariate logistic regression was performed to identify the factors associated with prediabetes and prehypertension.

**Results:**

Overall, the prevalence of prediabetes and prehypertension was 8.6% with 14% of the sampled population having from prediabetes and prehypertension separately. Among the prediabetic and prehypertensive participants, one-fourth of the participant were from the richest families and around one-third were overweight/obese, while more than fifty percent had normal Body Mass Index (BMI) and completed secondary and higher education. In the univariate analysis, the richest wealth status (UOR 3.3, 95% CI: 2.46 -4.35) and overweight/obesity (UOR 3.2, 95% CI: 2.62–3.85) are the highest predictors for prediabetes and prehypertension. After adjusting the other variables, overweight/obesity remains the largest predictor for prediabetes and prehypertension (AOR:2.5, 95% CI:2.05–3.05). Further, people aged 31 and above and from the richest family had around 2 times and 1.8 times higher risk of being prediabetic and prehypertensive compared to the younger age people (18–30 years) and the poorest family (respectively).

**Conclusion:**

The coexistence of prediabetes and prehypertension is an early sign of a greater burden of noncommunicable diseases (NCDs) in the near future for Bangladesh. To reduce the higher burden of NCDs, our findings call for a multisectoral approach to identify the precondition of NCDs with particular attention to maintaining body weight.

**Supplementary Information:**

The online version contains supplementary material available at 10.1186/s12889-023-16090-z.

## Background

Globally, diabetes and hypertension have been listed as the most prevalent non-communicable diseases (NCDs) and emerged as a serious public health concern [1]. South Asians are at greater risk to have type 2 diabetes, despite having low Body Mass Index (BMI) [2] and the incidence of diabetes among adults aged ≥ 45 years in South Asian men and women is 26 percent and 31.9 percent [3] whereas the prevalence of hypertension in adult ≥ 15 years is 27% [4]. It is alarming that, a significant number of individuals living with diabetes and hypertension remain unaware of their condition. For example, Around 8.8% of the adults having diabetes are undiagnosed [5] and the corresponding figure for hypertension is 46% [6].

Both Diabetes and hypertension are chronic diseases that requires long-term medical care [7] imposing a notable economic burden on individuals and their families and on the country’s health service system especially in resource-poor settings [8]. Also, the rapid flare-up of these diseases negatively impacts the poverty reduction initiatives in a country, particularly by increasing household costs associated with costs of healthcare along with the low family income (e.g. productivity loss from disability) [9].

Bangladesh, a country of 164 million people, has made remarkable progress in achieving socioeconomic development in every sector including the healthcare sector over the past few decades [10]. Because of ongoing demographic and epidemiological transition, the country is facing many significant public health challenges from dealing with infectious diseases to combating NCDs such as diabetes and hypertension. NCDs have substantially increased over the last decades in Bangladesh from ~ 5% in 2001 to ~ 14% in 2017, responsible for more than half of annual mortality (20–23). More than one-fourth adult aged 18 and more are hypertensive and one in ten adults are having higher blood glucose. Also, the prevalence of hypertension and higher blood glucose increases with increasing age and body mass index (BMI). In Bangladesh, about 61.5% of people are unaware that they have diabetes and half of the women and two third of the men are unaware that they have hypertension [11].

Prediabetes and prehypertension are far more common than frank diseases and are early warning signs of developing severe cardiovascular complications in seemingly healthy adults while they exist simultaneously [12]. Moreover, these preconditions are very crucial in their disease course because serious macro and microvascular complications present frequently at diagnosis that often fails to cure even after proper care has been taken [13, 14]. Therefore, early detection of disease by early screening opens the door to take prompt action for tackling any unexpected public health challenges. As the influencing factors for prediabetes and prehypertension shares common traits, a common effective prevention and control measures could be helpful in the optimization of NCDs management in Bangladesh [15]. Additionally, only by creating and raising awareness about adopting a healthy lifestyle at an early stage could prevent and/or delay the disease progression up to 30 to 67% in the future [16–19].

Nevertheless, the documentation of the rising prevalence of coexisting prediabetes and prehypertension is still very sparse and their hidden risk factors are still not well-acknowledged in Bangladesh. Following the release of Bangladesh Demographic and Health Survey (BDHS) data in 2011, a few studies examined the prevalence and determinants of prediabetes, diabetes, prehypertension, and hypertension [20, 21]. Another study compares the cross-country variation in the prevalence of prediabetes or diabetes along with hypertension and their determinants in rural South Asia (Bangladesh, Pakistan, and Sri Lanka). It also finds that prediabetes or diabetes is higher among hypertensive people in rural areas of South Asia [22]. Only one study assesses three chronic NCDs (prediabetes, diabetes, prehypertension, hypertension, and overweight/obesity) at a time using the BDHS’2011, paying particular attention to the differences in the area of residence and socio-economic inequality [23]. Using BDHS 2011, few studies have assessed the prevalence and contributing factors of prehypertension [21–24] and the studies are limited to age-standardized and stratified by sex. Also, the studies were limited to participants aged 35 years or more, leaving the likelihood of prevalence among younger adults not estimated and might be underestimated from the entire country’s point of view. Furthermore, the dataset used by the existing studies is no longer current data and was gathered more than eight years ago while first-time data on biomarkers were collected in the demographic health survey of Bangladesh. Also, BDHS 2017–18 collected information on 18 years and overpopulation and the studies used BDHS 2017–18 examine the prevalence and risk factors of diabetes, prediabetes, hypertension and prehypertension separately [25].

Because of the commonality in the predisposing factors for both prediabetes and prehypertension, common effective prevention and control measures could be helpful in the optimization of NCDs management in Bangladesh [15]. In order to reduce the future burden of diabetes and hypertension, it is very crucial to identify the important risk factors associated with these preconditions first. Also, detecting prediabetes and prehypertension at an earlier age is potentially beneficial, as this could guide specific management plans and potentially delay the disease process in young patients and help curb the risks of later life complications.

Therefore, the study aims to assess the current prevalence of coexisting prediabetes and prehypertension among Bangladeshi adults. In addition, we examine the determinants or influencing factors associated with prediabetes and prehypertension are examined by using the information on sociodemographic characteristics and measured biomarkers based on recent BDHS’2017–18 data.

## Methods

### Data

We used the eighth nationally representative cross-sectional survey BDHS’2017–18 dataset which was collected by the National Institute of Population Research and Training (NIPORT) with the collaboration of Mitra and Associates (Bangladesh) and ICF International (USA). A list of enumeration areas (EAs) known as primary sampling units (PSUs) from the 2011 National Population and Housing Census of Bangladesh provided by the Bangladesh Bureau of Statistics (BBS) had been used as a sampling frame. A two-stage stratified random cluster sampling method was followed in the BDHS’ 2017–18. In the first stage, 675 EAs (250 in urban areas and 425 in rural areas) were selected with probability proportional to EA size and a systematic sample of an average of 30 households per EA was selected for the enumeration in the second stage. Additionally, one-fourth of the households (5,063 households of 20,250 households) were randomly selected for blood pressure and fasting blood glucose measurements. All adults aged ≥ 18 years of the selected households were invited to participate and about 14,704 respondents participated for the biomarkers with a 90% response rate. Socio-demographic information including age, sex, marital status, education, current work status, occupation, anthropometry, individual possession of a mobile phone, asset information, and lifestyle factors were collected through different questionnaires. More details of the sampling procedure and data collection information are described elsewhere [11].

### Physical examination

In BDHS’2017–18, three biomarkers were incorporated: anthropometry (height and weight), blood pressure, and fasting blood glucose. A team of one male and one female health technicians collected the biomarker from the participants. After getting informed consent, weight was measured using lightweight, “electronic SECA 878 scales” with a digital screen, and height was taken with measuring boards made by Shorr Productions. Blood pressure (BP) was measured by the “LIFESOURCE® UA-767 Plus” digital BP monitoring machine. A total of three measurements of systolic and diastolic blood pressure (measured in millimeters of mercury [mmHg]) were taken during the interview at an interval of approximately 10 min. Then the average of the second and third measurements was used to report participant’s blood pressure values.

### Laboratory tests

Eligible participants for the measurement of the biomarkers were asked to fast overnight for 8 h and were visited early in the next day to ensure that they had not broken their fast before the test. In case, individuals who failed to take the test for any reason were requested again to fast overnight and perform the test on the following day. Blood specimen collection required at least two visits to the selected household for most of the cases. After confirming the overnight fasting, capillary whole blood was taken from the respondent’s middle or ring finger. The first two drops were wiped away, and then the third drop was taken for final measurement. The glucometer “HemoCue 201 RT analyzer” was used for the measurement and blood glucose values were documented in millimoles per liter (mmol/L).

### Study subject

**Fig. 1:**
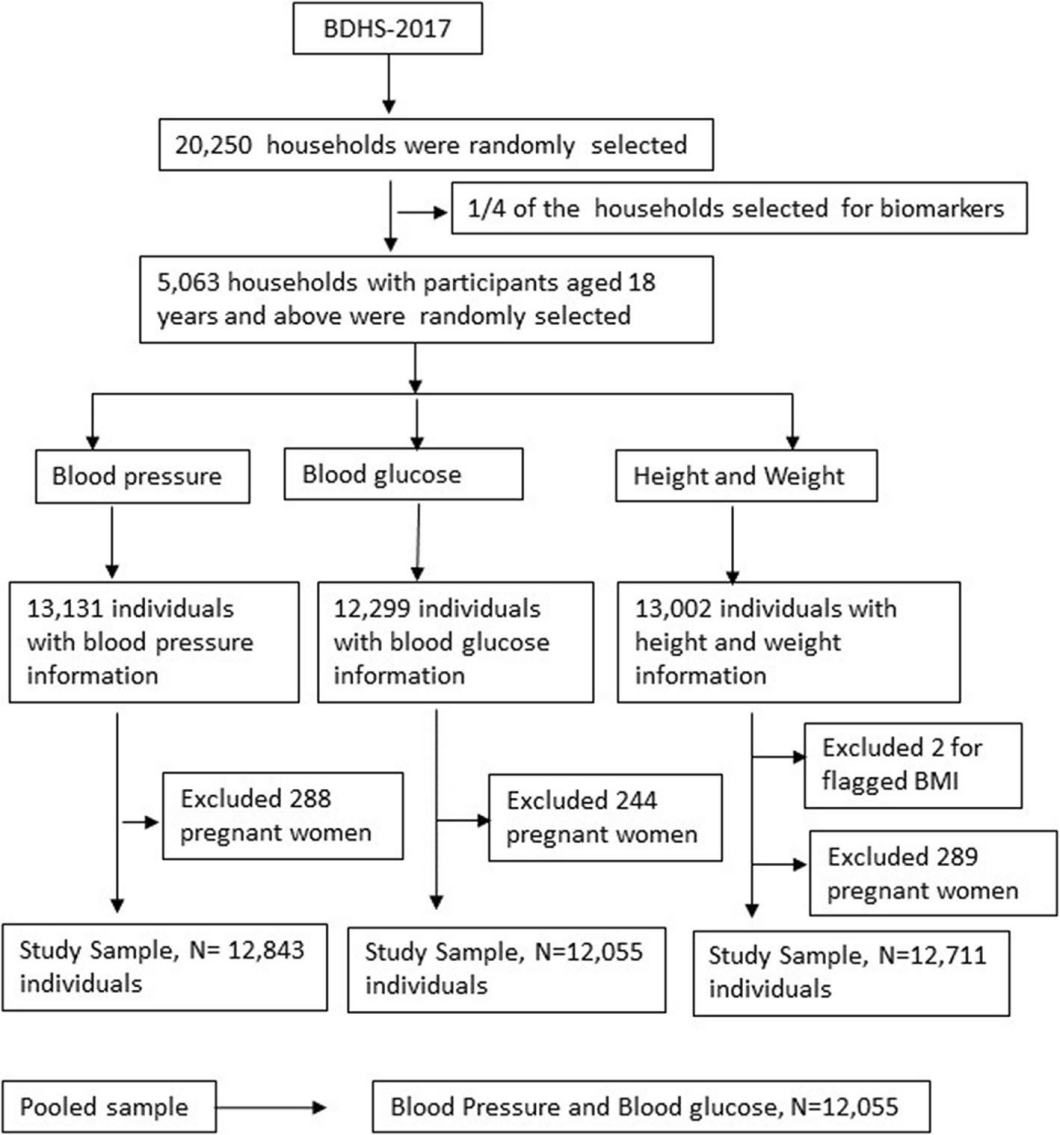
Sample selection procedure

Respondents who were having both blood glucose and blood pressure information in the BDHS’2017–18 were taken for this study. We have excluded pregnant women and got a total of 12,055 samples for this study. The sample selection procedure for this study is presented in Fig. 1.

### Dependent variables

Coexisting prediabetes and prehypertension is the main outcome variable of this study which is derived from the blood pressure (BP) and fasting glucose measurement. The definition and measurement criteria for the outcome variables are described in Table 1.

**Table 1:** Definition of dependent variables

Dependent variables	Diagnostic criteria	Reference
Prehypertension	If participants were the average systolic blood pressure (SBP) 130–139 mmHg AND/ OR average diastolic blood pressure (DBP) 80–89 mmHg was identified as the participant has prehypertension	JNC 7 [26]
Hypertension	If participants were with ≥ 140 mm Hg or above-average systolic blood pressure (SBP) or with ≥ 90 mm Hg or above-average diastolic blood pressure (DBP) or taking any anti-hypertensive medication in the survey time	JNC 7 [26]
Prediabetes	If a participant's FPG value within the range between 6.1 to 6.9 mmol/L (110–124 mg/dl) was classified as having prediabetic ((impaired FPG or intermediate hyperglycemia)	WHO [27]
Diabetes	If participants either had FPG levels equal to or more than 7 mmol/L (126 mg/dl) or reported having prescribed medicine for maintaining the blood glucose level	WHO [27]

### Independent variables

The sociodemographic characteristics such as participants’ age, gender, marital status, place of residence, occupation type, employment status, educational level, wealth quintile, smoking, and BMI, were included as potential influencing factors. The self-reported age variable was clustered into four different groups *i.e.,* ages between 18 − 30, 31 − 49, 50 − 64, and ≥ 65 years. We included eight administrative divisions namely Rajshahi, Barishal, Chattogram, Dhaka, Mymensingh, Khulna, Rangpur, and Sylhet of Bangladesh in the study, and the area of residence was divided into urban and rural. The educational level comprised no schooling or preschool, primary education, and secondary and higher education. The marital status of the participants was categorized into never married, married, and other (widowed and divorced). Following the standard demographic healthy survey wealth index method with the use of principal component analysis, BDHS’2017–18 provided the wealth index which, is further, categorized into five quintiles (poorest, poorer, middle, richer, and richest). Smoking was defined as smokers and non-smokers while employment status was defined as currently working or not. Different occupational categories were merged into manual and non-manual workers. The body mass index (BMI) was calculated as weight in kilograms divided by height in meters squared (kg/m2). BMI was categorized following the WHO guideline (Underweight: < 18.5 kg/m2, Normal: 18.5 kg/m2 to 24.9 kg/m2, Overweight: 25 kg/m2 to 29.9 kg/m2, and Obese: ≥ 30 kg/ m2) (49). For this study, BMI categories were merged into three categories underweight, normal, and overweight/obese.

### Statistical analysis

The descriptive statistics including mean, frequency, percentage, and Pearson’s Chi-Square test were performed for all exposure variables. Following the correlation among the variables of interest, we performed univariate and multivariate logistic regressions to find out the association between the predictor and outcome variables in different models. The unadjusted (UOR) and adjusted odds ratio (AOR), and *p*-values with 95% CI were presented. We adjusted the complex survey design using the survey weight provided by DHS for Bangladesh and the statistical software STATA version 17 was used for the data management and analysis.

## Results

In Table 2 we present the prevalence of prehypertension, hypertension, prediabetes, and diabetes among Bangladeshi adults. A total of 13,559 respondents, among them 3,076 participants are identified as having both normal blood pressure and blood glucose accounting for 22.5% of the total study population. On the contrary, a total of 1,127 cases have coexisting prediabetes and prehypertension together (accounting for 8.6% of the total study population). Further, approximately 14% of the total study population is having prediabetes and prehypertension separately. Also, among the perticipnats with coexisting prehypertension and prediabetes, we find the highest percentage of perticiapnts was living in rural area (69.5%) and Dhaka division (29%), and respondents who have higher educational attainment i.e. secondary/ higher education, and currently working as non-manual workers were corresponds with the higher proportion of coexisting diseases (46.8% and 62.2%, respectively). Further, around one-fourth of the participants of coexisting prediabetes and prehypertension was from the richest household and the proportion was 10 times higher in married participants compared to the unmarried participants (83.5% and 8.6% respectively). And, more than of half of the individuals with normal BMI had both prehypertension and prediabetes while it was around one third among overweight/obese participants of the study people with coexisting prediabetes and prehypertension (Table 3).

**Table 2:** Classification of prehypertension, hypertension, prediabetes, and diabetes in Bangladeshi Adults (*N* = 13,559), data drawn from the Bangladesh Demographic and Health Survey, BDHS 2017–18

**Type of Metabolism (n,%)**	N**ormal Blood Pressure**	**Prehypertension**	**Hypertension**	**Missing**	**Total (N)**
**Normal Blood glucose**	3,076 (22.5)	1,955 **(13.9)**	1,779 (12.6)	6 (0.04)	6,816 (48.9)
**Prediabetes**	1,777 **(13.9)**	1,127 **(8.6**)	1,109 (8.1)	0 (0.0)	4,013 (30.7)
**Diabetes**	324 (2.5)	305 (2.3)	594 (4.2)	3 (0.03)	1,226 (9.1)
**Missing**	362 (2.8)	226 (1.7)	215 (1.5)	701 (5.2)	1504 (11.3)
**Total (N)**	5,539 (41.8)	3,613 (26.4)	3,697 (26.5)	710 (5.3)	**13,559 (100.00**)

*n* number of respondents

*N* Total number of respondents

**Table 3:** Comparison and distribution of participants having normal, only prehypertension, only prediabetes, prediabetes and prehypertension, only hypertension, only diabetes, diabetes and hypertension among Bangladeshi adults aged 18 years and more drawn from the Bangladesh Demographic and Health Survey, 2017–18

Variables	Normal (n, %)	Only prehypertension	Only Prediabetes	Prediabetes & prehypertension	Only hypertension	Only diabetes	Diabetes & Hypertension
***Age***	***Mean age 39.74y***						***P*-value 0.000**
18-30y	1,559 (50.0)	920 (42.1)	843 (48.9)	378 (33.8)	486 (15.8)	168 (26.2)	38 (6.3)
31-49y	966 (31.5)	838 (38.3)	643 (35.2)	500 (44.6)	1,182 (38.3)	264 (41.9)	220 (37.7)
50-64y	356 (12.1)	294 (13.4)	203 (11.2)	170 (14.4)	818 (26.4)	140 (21.8)	206 (34.8)
> 65y	195 (6.4)	129 (6.2)	88 (4.7)	79 (7.2)	617 (19.5)	60 (10.0)	130 (21.2)
***Gender***							***P*-value 0.000**
Male	1,392 (44.7)	1,054 (48.2)	741 (40.8)	489 (43.9)	1298 (41.3)	297 (48.8)	238 (41.9)
Female	1,684 (55.3)	1,127(51.8)	1,036(59.2	638 (56.1)	1805 (58.7)	335 (51.2)	356 (58.0)
***Area of Residence***							***P*-value 0.000**
Rural	2,142(79.9)	1,383 (74.1)	1,105(68.2)	693 (69.5)	1980 (73.2)	360 (63.6)	311 (63.8)
Urban	934 (20.1)	798 (25.9)	672 (31.9)	434 (30.5)	1123 (26.9)	272 (36.4)	283 (36.2)
***Division***							***P*-value 0.000**
Rajshahi	447 (15.9)	331 (17.3)	199 (11.9)	114 (11.2)	404 (14.8)	75 (12.4)	64 (10.7)
Barisal	291 (5.2)	198 (4.9)	173 (4.8)	128 (5.4)	384 (6.9)	71 (5.6)	63 (5.3)
Chittagong	394 (17.0)	301 (17.8)	228 (15.2)	154 (16.7)	433 (17.9)	87 (16.1)	101 (22.4)
Dhaka	293 (18.0)	204 (17.1)	388 (35.3)	191 (29.0)	327 (18.9)	148 (37.7)	99 (28.9)
Khulna	416 (12.7)	332 (14.3)	204 (9.4)	171 (13.5)	447 (13.4)	63 (8.0)	96 (13.2)
Mymensingh	415 (10.1)	252 (8.3)	207 (8.1)	116 (7.3)	315 (7.3)	67 (7.0)	47 (5.9)
Rangpur	426 (13.8)	301 (13.9)	165 (8.8)	129 (10.9)	456 (14.5)	46 (6.8)	52 (7.3)
Sylhet	394 (7.2)	262 (6.5)	213 (6.5)	124 (5.9)	337 (6.2)	75 (6.5)	72 (6.3)
***Educational status***							***P*-value 0.000**
No Education/Preschool	685 (22.5)	506 (24.7)	375 (21.7)	260 (24.6)	1030 (35.0)	136 (23.4)	158 (27.6)
Primary	960 (31.3)	633 (29.1)	545 (29.8)	324 (28.5)	933 (29.4)	207 (33.2)	164 (28.0)
Secondary/higher	1,430 (46.2)	1,041 (46.2)	856 (48.5)	542 (46.8)	1140 (35.6)	288 (43.2)	272 (44.3)
***Working Status***							***P*-value 0.000**
No	1,134 (36.6)	726 (32.9)	661 (38.3)	438 (37.8)	1366 (43.1)	273 (42.6)	307 (51.1)
Yes	1,942 (63.4)	1,455 (67.1)	1,116 (61.7)	689 (62.2)	1737 (56.9)	359 (57.3)	287 (48.9)
***Occupation Type***							***P*-value 0.000**
Manual	1,337 (44.6)	898 (43.0)	706 (39.7)	394 (37.2)	1058 (35.9)	187 (31.8)	122 (20.7)
Not Manual	1,731 (55.2)	1,274 (56.8)	1,066 (59.4)	730 (62.4)	2037 (63.8)	443 (67.9)	471 (79.2)
***Wealth Index***							***P*-value 0.000**
Poorest	740 (23.5)	460 (21.8)	351 (18.3)	165 (15.1)	542 (18.1)	88 (13.0)	47 (8.3)
Poorer	716 (24.6)	430 (20.6)	327(18.1)	184 (16.7)	578 (19.6)	86 (13.8)	50 (9.3)
Middle	632 (21.5)	448 (22.6)	328 (18.9)	232 (20.6)	610 (20.6)	105 (16.5)	87 (15.9)
Richer	556 (18.2)	396 (17.3)	349 (20.9)	251 (22.9)	620 (20.0)	135 (23.9)	129 (21.2)
Richest	432 (12.3)	447 (17.7)	422 (23.8)	295 (24.6)	753 (21.7)	218 (32.8)	281 (45.3)
***Marital Status***							***P*-value 0.000**
Never Married	472 (14.7)	279 (11.6)	256 (13.4)	100 (8.6)	158 (4.4)	48 (7.2)	8 (1.7)
Married	2,401 (78.9)	1,739 (81.2)	1,400 (80.1)	937 (83.47)	2382 (77.5)	526 (84.7)	479 (82.0)
Others (separated, widow)	203 (6.4)	163 (7.2)	121 (6.6)	90 (8.0)	563 (18.1)	58 (8.1)	107 (16.3)
***Smoking Status***							***P*-value 0.000**
No	2,606 (85.9)	1,839 (84.4)	1,555 (88.6)	965 (86.9)	2535 (82.9)	525 (84.1)	491 (84.9)
Yes	468 (14.1)	342 (15.6)	222 (11.4)	162 (13.2)	567 (17.1)	104 (15.3)	103 (15.1)
***Body Mass Index (WHO)***							***P*-value 0.000**
Underweight	794 (25.7)	309 (14.7)	404 (22.7)	113 (10.2)	373 (11.8)	86 (14.9)	31 (5.7)
Normal	1,888 (61.5)	1,339 (60.6)	1,084(61.8)	658 (58.3)	1609 (51.5)	345 (54.8)	257 (44.4)
Overweight Obese	369 (11.9)	512 (23.7)	276 (14.8)	347 (30.7)	1055 (34.6)	193 (29.2)	287 (47.3)

^*^ X2 chi-square test **n = number of respondents

In Table 4 we represent both unadjusted and adjusted odds ratios for univariate and multivariate regression analyses of prediabetes with prehypertension group and normal group. In the univariate analysis, the richest wealth group (UOR 3.3, 95% CI: 2.46 -4.35) and overweight/obese (UOR 3.2, 95% CI: 2.62–3.85) are the largest predictors compared to the poorest and normal body weight groups. The age of adults between 31 and 49 years has 2.1 times higher odds (UOR 2.1, 95% CI: 1.75–2.45) of being prediabetic and prehypertensive than the age group below 30 years. Urban people are at 1.8 times higher risk of having prediabetes and prehypertension (UOR 1.8, 95%: 1.46–2.22), and the participants living in the Dhaka division have the greater odds (UOR 2.3, 95% CI: 1.56–3.46) than the other divisions.

**Table 4:** Univariate and Multivariate logistic regression analysis of prediabetes with prehypertension population comparing to and normal population

Variables	(Model-1)		(Model-2)	
	**Unadjusted OR**	**Unadjusted 95% CI**	**Adjusted OR**	**Adjusted 95% CI**
***Age***				
18-30 years	Ref *			
31-49 years	2.1***	1.755- 2.452	1.9***	1.626—2.335
50-64 years	1.7***	1.360—2.174	2.1***	1.646—2.723
> 65 years	1.6	1.176- 2.179	2.0***	1.377—2.796
***Gender***				
Male	Ref			
Female	1	0 .887—1.201		
***Area of residence***				
Rural	Ref			
Urban	1.8***	1.462- 2.221	1.2	0 .977—1.533
***Division***				
Rajshahi	Ref			
Barisal	1.4	0.983—2.110	1.5*	1.022—2.110
Chittagong	1.4	0 .933—1.960	1.2	0.829—1.703
Dhaka	2.3***	1.566 -3.4661	1.8**	1.231—2.742
Khulna	1.5*	1.048—2.059	1.4	0.983—1.921
Mymensingh	1	0.719—1.451	1.2	0.821—1.622
Rangpur	1.1	0.774—1.591	1.3	0.909—1.863
Sylhet	1.2	0.803—1.660	1.2	0.841—1.730
***Educational status***				
Secondary/ Higher	Ref			
Primary	0.9	0 .894—1.305		
No education/ Preschool	1.1	0.752—1.083		
***Currently employed***				
No	Ref			
Yes	0.9	0 .799—1.129		
***Occupation Type***				
Manual	Ref			
Not Manual	1.4***	1.141—1.596	1.2	0.994—1.393
***Wealth status***				
Poorest	Ref			
Poorer	1.1	0.793—1.378	0.9	0.722—1.234
Middle	1.4**	1.093 1.873	1.2	0.934—1.608
Richer	1.9***	1.436- 2.516	1.4*	1.048- 1.821
Richest	3.3***	2.458—4.349	1.8**	1.306- 2.424
***Marital Status***				
Never Married	Ref			
Married	1.8***	1.390–2.357	1.1	0.856- 1.485
Others (divorced/ widow)	2.2***	1.539–3.108	1.2	0.798- 1.793
***Smoking Status***				
No	Ref			
Yes	0 .9	.732- 1.164		
***BMI (WHO)***				
Normal	Ref			
Underweight	0.5***	0 .409- .639	0.5***	0.429—0.679
Overweight/Obese	3.2***	2.624- 3.855	2.5***	2.057- 3.053

^*^p < 0.05; **p < 0.01; ***p < 0.001 *The total, N, varies from variable to variable because of missing data. * *OR* Odds ratio, *CI* Confidence intervals, Ref reference  category

In the multivariate analysis, BMI remains the biggest significant factor even after controlling for other variables and overweight/obese being prediabetic and prehypertensive (AOR:2.5, 95% CI:2.05–3.05). People from the Dhaka division (AOR 1.8, 95% CI: 1.23- 2.74) and richest wealth group (AOR 1.8, 95% CI: 1.30- 2.42) show a significant association with a higher likelihood of having both prediabetes and prehypertension compared to the Rajshahi division and the poorest family. Also, people aged 31 years and above are having around 2 times higher risk of having prediabetes and prehypertension compared to younger age people (18–30 years).

## Discussion

To the best of our knowledge, this is the first national-level study in Bangladesh that assessed the prevalence and predictors associated with prediabetes and prehypertension simultaneously by using the most recent country-representative dataset. Overall, approximately one out of ten individuals (8.6%) aged 18 years and older are having prediabetes and prehypertension. Among the participants with prediabetes and prehypertension, the highest proportion is found among people aged 30 to 49 years (44.65%) while one-fourth of the participants who have prediabetes and prehypertension are from the richest family.

Studies that used BDHS’2017–18 reported that the age-standardized prevalence of prediabetes is 14% and the pooled prevalence of prediabetes is 10.1% which is similar to our study findings for prediabetes (13.9%) [28, 29]. Additionally, the overall prevalence of coexisting prediabetes and prehypertension among Chinese adults is 11% which was quite close to our study findings [30, 31]. This emplies that the trend of prediabetes and prehypertension in two of the populous country is same. The prevalence from our study for prediabetes and prehypertension is 8.6% which is equivalent to 14 million individuals in 2020. This large number of cases in Bangladesh underlines the urgent need for policies supporting the rollout of prevention strategies in the country.

Evidence suggests that prediabetes and prehypertension is a major concern among older adults aged 35 years and more [20, 25, 29, 32–35]. We also find a significant positive association between age and increasing risk of prediabetes and prehypertension in Bangladesh and the probability of having prediabetes with prehypertension is double for the older age groups compared to the younger age group. However, recently an increasing trend in the prevalence of prediabetes and prehypertension conditions is commonly found among younger adults aged less than 35 years because of ongoing epidemiologic and demographic transition [25, 28, 36]. In our study, one-third of the younger participants (18 to 30 years) are prediabetic and prehypertensive. Earlier studies find the prevalence of prediabetes with prehypertension among young adults is not as low as generally predicted while these individuals are often neglected, have a lower level of awareness, poor adherence to physical activity, smoking habits, and worse weight regulation power [37–39]. Also, around 40.5% of individuals with prediabetes convert to diabetes during subsequent follow-up [40]. The high conversion rate indicates that most of the population aged between 18 to 34 years are at greater risk to be diabetic or hypertensive in the coming years while age is considered a non-modifiable risk factor potential for an uncontrolled increase [41].

Prehypertension and prediabetes are found to have a significant association with the place of residence, particularly urban areas, [28] which is consistent to our study findings. Higher socioeconomic status and lower physical activity of the people living in the urban areas might be the contributing factors to the higher prevalence of prehypertension and prediabetes [42]. Additionally, the findings suggest that there was a striking variation across the geographical regions within the country. For example, the highest prevalence of prediabetes and prehypertension was observed in the central Dhaka division (29%), followed by the western Khulna division (13%) and the northwestern part Rangpur division (10%). An earlier study found a higher risk of people living in the Dhaka division had greater odds 1.3 times of being prehypertensive with prediabetic [28] which supports the study findings. This difference may be due to factors like more intake of fast food, stressful life, urbanized transport system, lack of opportunity to physical exercise and lack of awareness of early detection are prevalent in urban areas like Dhaka city [43]. In contrast, the Rangpur devison is less urbanized compared to Dhaka which might explain the low prevalence rate in the Rangpur division. Thus, it is important to prioritize the higher prevalence areas with appropriate preventive programs.

The study finds a significant relationship between respondents’ wealth status and the coexistence of prehypertension and prediabetes. Among survey participants, well-off people are more likely to be prehypertensive and prediabetic. This finding is consistent with other studies that investigated the prevalence and risk factors of prehypertension in Bangladesh and other South Asian countries [22]. Wealth status is found to have an influence on the behavioral factors of an individual [21, 28]. Because of having higher wealth, people of higher socioeconomic status in low and middle-income countries (LMICs) adopt a more sedentary lifestyle and are capable of availing more consumable resources than their lower socioeconomic counterparts which might contribute to the higher prevalence of prehypertension and prediabetes [44]. Therefore, increasing awareness and adoption of a healthier lifestyle is crucial for high-risk groups.

Studies find that higher BMI (overweight/obesity) is an independent and robust predictor of developing prediabetes and prehypertension [4, 22, 28, 45–48] which is align with our study findings. The likelihood of developing coexisting prediabetes and prehypertensive was much higher among individuals who have higher waist circumference and BMI [26]. In our study, overweight/obese participants are having 2.5 times higher risk of being prediabetic and prehypertensive compared to the normal weight participants. Overweight and obesity has increased in Bangladesh over the last of couple years, increasing from 12 to 32% for women aged 15–49 years between 2007 to 2017. The rate of overweight and obesity is 18% for men aged 18 years and more and it is the highest (24%) among people aged 30–39 years [11]. Ongoing demographic and nutritional transition i.e. rapid urbanization, uncontrolled economic development, and globalization of unhealthy lifestyles especially among the younger generation could be attributed to the increasing BMI in Bangladesh [27]. In addition, the greater consumption of unhealthy processed food and lack of physical activity has an impact on increasing BMI [49, 50]. The increasing trend of overweight and obesity in Bangladesh suggests that the prevalence of prediabetes and prehypertension will increase further. Urgent policy and program implication for the prevention of diabetes and hypertension is needed with focusing on the reduction of overweight and obesity in Bangladesh.

We did not find any significant relationship between the coexistence of prediabetes and prehypertension and the level of education of the participants, and the finding is aligned with the other studies that used BDHS’2011 and BDHS’2017–18 for examining the prehypertension and prediabetes separately. Another study conducted in Iran did not find a significant association between prediabetes and education for both women and men in the Iranian Urban Population [51]. Nevertheless, because of race/ethnicity and various local factors (e.g. climate change and lifestyle), the prevalence of prehypertension and prediabetes varies considerably in different countries [52].

The present study has several notable strengths. The study uses the most updated BDHS’2017–18 survey which covers the younger participants aged 18–34 years and more with a large sample size compared to the BDHS’2011. As the BDHS’2017–18 is a representative of rural, urban and all administrative divisions of Bangladesh, the robust study findings have made the results generalizable (i.e. external validity) to all regions of Bangladesh. Following the standard procedure, expert health technicians collected the biomarker information (blood pressure and blood glucose). Also, the study utilizes widely validated research procedures with an advance statistical method that further increases the authenticity of the study findings. However, BDHS’2017–18 is a cross-sectional survey, we are not able to examine the causal relationship between the dependent and independent variables. Fasting blood glucose (FBG) was not complemented with an oral glucose test (OGTT) or glycosylated HbA1c test. Blood Pressure was recorded in a single setting, but longitudinal measurement is recommended to confirm the diagnosis [53, 54]. Also, a few important known risk factors including genetic predisposition of diabetes and hypertension, psychological stress, lifestyle, pattern of physical activity, dietary habit (e.g., salt intake), measures of central obesity i.e. waist circumference, hip circumference, and their ratio, blood HDL-cholesterol levels and exposure to other drugs, insulin resistance and beta-cell function were not collected in the BDHS’2017–18.

The study has policy implications that could be guided the policymakers and relevant stakeholders to design specific treatment and lifestyle management strategies for young adults in Bangladesh to avert the negative consequences associated with NCDs. The study findings could be a piece of evidence to develop age-specific management strategies, especially targeting young adults aged 18–34 years, as the National Guidelines for management of hypertension and diabetes in Bangladesh focuses on country-specific guidelines for effective management only [55, 56]. Also, a significant proportion of prediabetes and prehypertensive are likely to develop full hypertension and diabetes in the near future. Therefore, the findings of this study highlight the significance of screening and detecting prediabetes and prehypertension at an early stage to minimize the burden of NCDs in Bangladesh. Evidence suggests that young people are more likely to be less concerned and reluctant in timely BP measurement and blood sugar monitoring thus, interventions should be directed to encourage young adults to an earlier screening approach [25, 57].

## Conclusion

In conclusion, the study finds a high prevalence of prehypertension and prediabetes among younger and middle-aged people. Overweight/obesity, higher wealth status, urban areas and non-manual occupation are the major risk factors for both prediabetes and prehypertension. Also, people aged more than 30 years are at greater risk to have prediabetes and prehypertension compared to younger aged (18–30 years) people. Although age is an un-modifiable factor, care should be taken among these populations emphasizing the preventive interventions on modifiable behavioral factors such as weight reduction, encouraging physical activity, and promoting a healthy diet. To achieve the WHO’s Global NCD Monitoring Framework’s target of one-third reduction in premature deaths from cardiovascular disease by the year 2025, a comprehensive and predictive community-based approach is highly needed with aiming at the young generation for Bangladesh.

## Supplementary Information

**Additional file 1:** **Appendix 1.** Univariate & Multivariate logistic regressionanalysis of diabetes with hypertension group and normal   group. **Appendix 2.** Univariate & Multivariate logistic regressionanalysis of diabetes with hypertension group and prediabetes withPrehypertension group.

## Data Availability

The dataset used for this study is publicly for the researchers from the Demography and Health surveys (https://dhsprogram.com/Countries/Country-Main.cfm?ctry_id=1&c=Bangladesh&Country=Bangladesh&cn=&r=4).

## Abbreviations

NCDs: Non-Communicable Diseases
BDHS: Bangladesh Demographic and Health Survey
LMICs: Low and Middle Income Countries
FBG: Fasting Blood Glucose
BP: Blood Pressure
GDP: Gross Domestic Product
WHO: World Health Organization
BMI: Body Mass Index
CI: Confidence Interval
OR: Odd Ratio
UOR: Unadjusted Odd Ratio
AOR: Adjusted Odd Ratio
NIPORT: National Institute for Population Research Training
