# Supplementary material for: The prevalence and influencing factors of coexisting prediabetes and prehypertension among Bangladeshi adults
Source: BMC Public Health. 2023 Jun 20;23:1184. doi: 10.1186/s12889-023-16090-z (PMC10280828; doi:10.1186/s12889-023-16090-z)
Supplement: Supplementary file 1 — Additional file 1: Appendix 1. Univariate & Multivariate logistic regressionanalysis of diabetes with hypertension group and normal group. Appendix 2. Univariate & Multivariate logistic regressionanalysis of diabetes with hypertension group and prediabetes withPrehypertension group. [file 12889_2023_16090_MOESM1_ESM.docx]

**Appendix 1:** Univariate & Multivariate logistic regression analysis of diabetes with hypertension group and normal   group.

| ***Variables*** | ***Model 1*** | | ***Model 2*** | |
| --- | --- | --- | --- | --- |
|  | ***Unadjusted OR*** | ***Unadjusted 95% CI*** | ***Adjusted OR*** | ***Adjusted 95% CI*** |
| ***Age*** | | | | |
| 18-30years | Ref |  |  |  |
| 31-49years | 3.3*** | 2.691- 4.148 | 3.0*** | 2.360- 3.803 |
| 50-64years | 11.8*** | 9.442- 14.69 | 10.7*** | 8.219-13.822 |
| >65years | **14.9***** | 11.070-19.93 | **13.3***** | 9.352-18.986 |
| ***Gender*** | | | | |
| Male | Ref |  |  |  |
| Female | 1.1 | 0.9163- 1.362 |  |  |
| ***Area of residence*** | | | | |
| Rural | Ref |  |  |  |
| Urban | **2.5***** | 2.001 -3.157 | 1.2 | 0.915- 1.505 |
| ***Division*** | | | | |
| Rajshahi | Ref |  |  |  |
| Barisal | 1.4 | 0.871- 2.386 | 1.3 | 0.900- 2.008 |
| Chittagong | 1.9** | 1.260 -2.920 | 1.2 | 0.796- 1.706 |
| Dhaka | **2.5***** | 1.586 - 3.834 | 1.3 | 0.920- 1.963 |
| Khulna | 1.5 | 0 .971- 2.254 | 1.1 | 0.758- 1.668 |
| Mymensingh | 0.9 | 0.570 - 1.442 | 1.0 | 0.707- 1.463 |
| Rangpur | 0.9 | 0.546 - 1.329 | 1.1 | 0.732- 1.541 |
| Sylhet | 1.3 | 0.779- 2.003 | 1.1 | 0.740- 1.599 |
| ***Educational status*** | | | | |
| Secondary/ Higher | Ref |  |  |  |
| Primary | 0.9 | 0.737 - 1.19 |  |  |
| No education /Preschool | 1.3 | 1.014- 1.661 |  |  |
| ***Working Status*** | | | | |
| No | Ref |  |  |  |
| Yes | 0.5*** | 0.0444 - .6679 | 1.1 | 0.816- 1.378 |
| ***Occupation Type*** | | | | |
| Manual | Ref |  |  |  |
| Not Manual | 2.7*** | 2.158 -3.375 | 1.7*** | 1.312- 2.194 |
| ***Wealth Index*** | | | | |
| Poorest | Ref |  |  |  |
| Poorer | 1.0 | 0.714 - 1.475 | 0.9 | 0.624-1.166 |
| Middle | 1.6 | 1.081- 2.277 | 1.2 | 0.903-1.685 |
| Richer | 2.4*** | 1.681- 3.45 | 1.5** | 1.093- 2.143 |
| Richest | **13.5***** | 9.609-18.897 | 4.2*** | 2.914- 6.021 |
| ***Marital Status*** | | | | |
| Never Married | Ref |  |  |  |
| Married | 2.9*** | 2.184- 3.906 | 1.1 | 0.783- 1.407 |
| Others | **9.7***** | 6.789-13.80 | 1.3 | 0.840 -1.988 |
| ***Smoking Status*** | | | | |
| No | Ref |  |  |  |
| Yes | 1.1 | 0.820- 1.441 |  |  |
| ***BMI (WHO)*** | | | | |
| Normal | Ref |  |  |  |
| Underweight | 0.6*** | 0.421- 0.712 | 0.5*** | 0.434 - 0.684 |
| Overweight/  Obese | **10.4***** | 8.226-13.192 | 5.3*** | 4.171- 6.744 |

 *p<0.05; **p<0.01; ***p<0.001 p-value adjusted for survey design. *The total, N, varies from variable to variable because of missing data. *OR odds ratio, CI confidence intervals, Ref referent category

**Appendix 2:** Univariate & Multivariate logistic regression analysis of diabetes with hypertension group and prediabetes with Prehypertension group.

| ***Variables*** | ***Model 1*** | | ***Model 2*** | |
| --- | --- | --- | --- | --- |
|  | ***Unadjusted OR*** | ***Unadjusted 95% CI*** | ***Adjusted OR*** | ***Adjusted 95% CI*** |
| ***Age*** | | | | |
| 18-30years | Ref |  |  |  |
| 31-49years | 2.6*** | 1.891 -3.543 | 2.8*** | 1.983 – 4.020 |
| 50-64years | 8.0*** | 5.627-11.322 | 9.1*** | 6.215 -13.349 |
| >65years | **10.0***** | 6.757-14.687 | **12.1***** | 7.701- 19.101 |
| ***Gender*** | | | | |
| Male | Ref |  |  |  |
| Female | 1.1 | 0.858 -1.364 |  |  |
| ***Area of residence*** | | | | |
| Rural | Ref |  |  |  |
| Urban | 1.3* | 1.012 -1.667 | 0.9 | 0.625 -1.181 |
| ***Division*** | | | | |
| Rajshahi | Ref |  |  |  |
| Barisal | 1.0 | 0.630 -1.668 |  |  |
| Chittagong | **1.4** | 0.930 -2.210 |  |  |
| Dhaka | 1.0 | 0.665 -1.651 |  |  |
| Khulna | 1.0 | 0.653 -1.629 |  |  |
| Mymensingh | 0.9 | 0.504 -1.462 |  |  |
| Rangpur | 0.7 | 0.436 -1.197 |  |  |
| Sylhet | 1.1 | 0.656 -1.882 |  |  |
| ***Educational status*** | | | | |
| Secondary/ Higher | Ref |  |  |  |
| Primary | 1.0 | 0.793 -1.352 |  |  |
| No education/  Preschool | 1.2 | 0.894 -1.580 |  |  |
| ***Working Status*** | | | | |
| No | Ref |  |  |  |
| Yes | 0.6*** | 0.462 -0.730 | 1.0 | 0.725 -1.292 |
| ***Occupation Type*** | | | | |
| Manual | Ref |  |  |  |
| Not Manual | 2.4*** | 1.812 -3.104 | 1.7** | 1.189 – 2.407 |
| ***Wealth Index*** | | | | |
| Poorest | Ref |  |  |  |
| Poorer | 1.0 | 0.633 -1.585 | 1.0 | 0.600 -1.526 |
| Middle | 1.3 | 0.842 -2.071 | 1.2 | 0.763 - 1.908 |
| Richer | 1.6 | 1.030 -2.361 | 1.3 | 0.840 - 2.032 |
| Richest | **3.3***** | 2.186 -4.839 | 2.5*** | 1.566 - 3.896 |
| **Marital Status** | | | | |
| Never Married | Ref |  |  |  |
| Married | 3.4*** | 1.875-6.100 | 1.0 | 0.516 - 1.771 |
| Others | **8.3***** | 4.356-15.726 | 1.4 | 0.580 - 2.678 |
| **Smoking Status** | | | | |
| No | Ref |  |  |  |
| Yes | 1.2 | 0.861 -1.617 |  |  |
| **BMI (WHO)** | | | | |
| Normal | Ref |  |  |  |
| Underweight | 0.8 | 0.502 -1.201 | 0.8 | 0.498 - 1.186 |
| Overweight/ Obese | **2.1***** | 1.638-2.590 | 1.8*** | 1.396 -2.362 |

*p<0.05; **p<0.01; ***p<0.001 p-value adjusted for survey design

*The total, N, varies from variable to variable because of missing data. *OR odds ratio, CI confidence intervals, Ref referent category.
